# Human trophoblast invasion: new and unexpected routes and functions

**DOI:** 10.1007/s00418-018-1699-0

**Published:** 2018-07-26

**Authors:** Gerit Moser, Karin Windsperger, Jürgen Pollheimer, Susana Chuva de Sousa Lopes, Berthold Huppertz

**Affiliations:** 1 Department of Cell Biology, Histology and Embryology, Gottfried Schatz Research Center, Medical University of Graz, Neue Stiftingtalstrasse 6/II, 8010 Graz, Austria; 2 Division of Obstetrics and Feto-maternal Medicine, Department of Obstetrics and Gynecology, Medical University of Vienna, Vienna, Austria; 3 Reproductive Biology Unit, Department of Obstetrics and Gynecology, Medical University of Vienna, Vienna, Austria; 4 Department of Anatomy and Embryology, Leiden University Medical Center, Leiden, The Netherlands; 5 Department for Reproductive Medicine, Ghent University Hospital, Ghent, Belgium

**Keywords:** Extravillous trophoblast, Endoarterial trophoblast, Endovenous trophoblast, Endovascular trophoblast, Endoglandular trophoblast, Endolymphatic trophoblast, Invasion, Placenta, Recurrent spontaneous abortion (RSA), Tubal pregnancy

## Abstract

Until recently, trophoblast invasion during human placentation was characterized by and restricted to invasion into uterine connective tissues and the uterine spiral arteries. The latter was explained to connect the arteries to the intervillous space of the placenta and to guarantee the blood supply of the mother to the placenta. Today, this picture has dramatically changed. Invasion of endoglandular trophoblast into uterine glands, already starting at the time of implantation, enables histiotrophic nutrition of the embryo prior to perfusion of the placenta with maternal blood. This is followed by invasion of endovenous trophoblasts into uterine veins to guarantee the drainage of fluids from the placenta back into the maternal circulation throughout pregnancy. In addition, invasion of endolymphatic trophoblasts into the lymph vessels of the uterus has been described. Only then, invasion of endoarterial trophoblasts into spiral arteries takes place, enabling hemotrophic nutrition of the fetus starting with the second trimester of pregnancy. This new knowledge paves the way to identify changes that may occur in pathological pregnancies, from tubal pregnancies to recurrent spontaneous abortions.

## Introduction

The last years have seen a massive evolution of omics and sequencing technologies that finally allow single-cell sequencing and profiling. Single-nucleus RNA sequencing per droplet (DroNc-Seq) (Habib et al. 2017), single-cell combinatorial indexing RNA sequencing (sci-RNA-seq) (Cao et al. 2017) and other sequencing technologies open new avenues to analyze DNA/RNA on a single-cell level. At the same time, all these new methodologies do not help in achieving information about the localization of a single cell within a complex tissue. This has become clear now and has been especially recognized in the new “Human Cell Atlas” project that started in 2017. This project is based on the creation of a comprehensive reference map of all human cells, which is only as good as the histological and morphological classification of cells within a tissue. The information on the RNA profile of a cell is only of value if the spatial localization of this cell including the micro-environmental context is available at the same time.

Hence, there is the need to combine both fields, sequencing and histology, to enable an even more comprehensive view on the distribution and localization of specific cell types within a tissue on the RNA level. Respective technologies are already available such as the detection of RNA species using padlock probes and the rolling circle amplification (Mezger et al. 2014). A first report on the use of this technology on placental tissues has already been published (Siwetz et al. 2016). Such methodologies will surely enable a much broader view on expression patterns of RNA species on cells with known phenotype. This can then be directly linked to the micro-environment of these cells.

Focusing on the morphological aspects of placentation in the human, we have revisited the notion that the placental trophoblast only invades into uterine arteries while ignoring all other luminal structures in the uterine wall. Interestingly, it is general knowledge since 60 years and longer that uterine veins are connected to the placenta and drain back maternal blood that reaches the intervillous space of the placenta via invaded spiral arteries. The iconic schematic drawings of Elisabeth Ramsey depict well the changes of uterine arteries and veins during pregnancy and show how the placenta is connected to the maternal vasculature (Ramsey 1959). Only very recently, invasion of extravillous trophoblast into uterine veins has been detected (He et al. 2017; Moser et al. 2017; Windsperger et al. 2017).

Similarly, the nutritional feeding of the embryo with secretion products of the uterine glands (uterine milk) has been proposed in 2002 (Burton et al. 2002). Again, the answer to the question, how such secretion products may reach the intervillous space of the placenta has only been provided in 2010 (Moser et al. 2010).

Details of the invasive pathways of the extravillous trophoblast during early pregnancy will be shown and discussed in this review.

## Migratory routes of extravillous trophoblast in normal pregnancy

With the availability of HLA-G-specific antibodies, a much better assessment of trophoblast invasion was possible, especially in the first trimester of pregnancy. Previously, the assessment had mostly been performed with antibodies against different cytokeratin isotypes, mostly against cytokeratin 7 (KRT7). The disadvantage of using a cytokeratin antibody is that it also identifies other epithelial cells such as the epithelium of uterine glands. Hence, a missing differentiation between invading trophoblasts and glandular epithelial cells may have misled scientists (Moser et al. 2011). At the same time, the antibody against HLA-G should be chosen with caution as only HLA-G1 is expressed on the cell surface of extravillous trophoblasts (Moser et al. 2011).

### Interstitial trophoblast invasion

Looking at the invasion of trophoblasts into the uterine wall once the placenta proper has developed, the trophoblastic cell columns at the bases of the anchoring villi are the predominant source of invading trophoblasts (Kaufmann et al. 2003). Placental villi connected to the basal plate are called anchoring villi that help in attaching and anchoring the placenta proper to the uterine wall. At the sites where the villous surface comes into contact with the maternal stroma, the syncytiotrophoblast degenerates and the underlying cytotrophoblasts start to proliferate and generate cell columns.

**Fig. 1:**
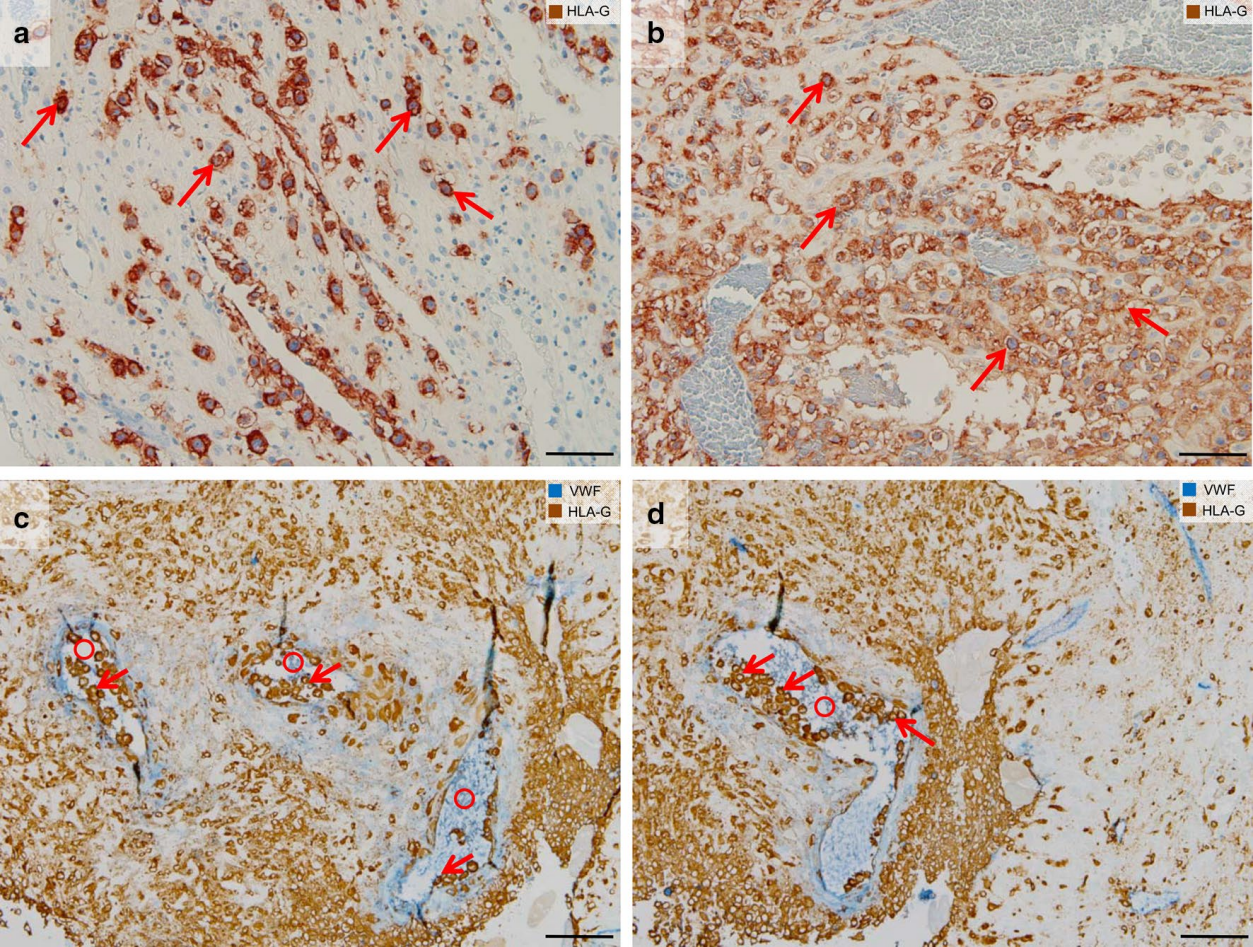
Interstitial and endoarterial trophoblast invasion. Immunohistochemical single (**a, b**) and double staining (**c, d**) of paraffin sections from the first trimester decidua basalis (gestational age 7–8 weeks). **a, b **The first row shows images stained for HLA-G (brown, extravillous trophoblasts) and counterstained with hemalaun. **c, d** The second row shows images stained for HLA-G (brown, extravillous trophoblasts) and von Willebrand factor (vWF, blue, vascular endothelium) without nuclear counterstain. **a, b** Interstitial trophoblasts (arrows show examples) are situated in the decidual stroma. **c, d** Endoarterial trophoblasts (arrows) accumulate to trophoblast plugs in the lumen of spiral arteries (circles). Scale bars represent 50 µm in **a, b** and 100 µm in **c, d**

The proliferative pressure of the cytotrophoblasts resting on the basement membrane of the anchoring villi pushes the post-proliferative daughter cells toward the uterine wall underneath the placenta, the placental bed. With the loss of contact to the basement membrane, the daughter cells change their phenotype during their movement through the cell columns. They change their repertoire of matrix metalloproteinases (MMPs) (Huppertz et al. 1998a) and start expressing a specific type of the human leukocyte antigen, HLA-G (McMaster et al. 1995). Finally, the cells come into direct contact with the uterine tissues and start their invasive pathway into the interstitium of the uterus (Fig. 1). This is why this type of extravillous trophoblast is termed interstitial trophoblast (Benirschke et al. 2012).

Different from monkey, the human placenta does not develop a fully grown trophoblast shell (Burton and Jauniaux 2017). This shell is only present for a short time interval very early in pregnancy. It then dissolves and single cell columns remain as sites for trophoblast invasion. This difference is important for the identification of the routes of trophoblast invasion. At the time of the trophoblast shell, trophoblast cells may directly enter the lumen of vessels that have already been opened interstitially. Afterward, trophoblast cells need to take the interstitial route of invasion from the cell columns to reach new arteries and veins and/or deeper parts of the vessels (Kaufmann et al. 2003). Hence, the interstitial trophoblast population is the source and gives rise to all other trophoblast populations described below. At the same time, the interstitial trophoblast has a function of its own, which is attaching and adhering the placenta to the uterine wall. This is achieved by secretion of a specific extracellular matrix, called matrix-type fibrinoid (Kaufmann et al. 1996) that can be found around remaining interstitial trophoblast even years after delivery (Huppertz, unpublished).

### Endoarterial trophoblast invasion

So far, it is still unclear whether the different subpopulations of extravillous trophoblast that invade into luminal structures are already predefined in the uterine interstitium or whether they just invade into the next luminal structure without any specific route of invasion. The type of invasion into arteries is generally different compared to invasion into veins and glands. Glands and veins are invaded to be connected to the intervillous space of the placenta without the need of a massive transformation of their walls. For arteries, veins and glands, there is real functional significance to be invaded as they need to be connected to the intervillous space of the placenta. This significance still needs to be shown for invasion into uterine lymph vessels.

Invasion of endoarterial trophoblasts into spiral arteries shows similarities to invasion into veins and glands (A), but also differs in a variety of features (B–E):

(A) Interstitial trophoblasts invade through the uterine interstitium, finally reaching the outer aspects of the arterial wall. Endoarterial trophoblasts percolate through the vessel wall, restructure the arterial media, displace the arterial endothelium and finally migrate into the lumen of the arteries (Kaufmann et al. 2003).
(B) During the first trimester, the endoarterial trophoblasts do not only reach and line the inner wall of the arteries, but also fill the lumen by building plugs of endoarterial trophoblasts (Weiss et al. 2016). This leads to blockage of the vessel lumen hindering flow of maternal blood cells into the intervillous space of the placenta. The plugs only allow passage of plasma plus some smaller corpuscular particles up to the size of platelets (Roberts et al. 2017; Weiss et al. 2016). Robert et al. (2017) have used lipid-shelled microbubbles to identify flow from the maternal vascular system into the placenta. The authors could show that the microbubbles that had a similar size (1.1–3.3 µm) as compared to platelets (max 2–3 µm) were able to pass the plugs already at week 6 of pregnancy.
(C) Different from veins and glands, arterial invasion by endoarterial trophoblasts leads to transformation of the vessel walls resulting in large conduits that are no longer under vasomotor control of the mother.
(D) Different from veins and glands, flow of maternal blood through arteries into the intervillous space of the placenta only starts at the beginning of the second trimester (Jauniaux et al. 2000). This secures hemotrophic nutrition of the fetus until delivery.
(E) Different from veins and glands, endoarterial trophoblasts also invade into the deeper regions of the arterial walls, finally reaching the portions of the arteries within the inner third of the myometrium. Veins and glands only need to be connected to the intervillous space of the placenta, while arteries need to be connected plus transformed and widened to enable a slow inflow of maternal blood into the intervillous space (Burton et al. 2009).

### Endovenous and endolymphatic trophoblast invasion

The vascular system consists of two main parts: the circulatory system transporting blood through arteries and veins toward heart and lungs as well as toward end organs; and the lymphatic system, a unidirectional system carrying lymph fluid toward the heart and fusing with the bloodstream via the subclavian veins. While the main function of the circulatory system is to provide a constant supply of nutrients and oxygen to the cells of the body and to remove waste products, the lymphatic system is responsible for the fluid balance in the body, reabsorbing the interstitial fluid that leaks from the circulatory system. In addition, the lymphatic system serves another important purpose: transporting lymphocytes. It is connected with lymph nodes and lymphatic organs such as spleen, thymus, tonsils and the bone marrow. In the human, the first lymphatics develop around week 5 of development (week 7 of pregnancy) from the subclavian veins in the junction with the jugular veins (van der Putte and van Limborgh 1980).

**Fig. 2:**
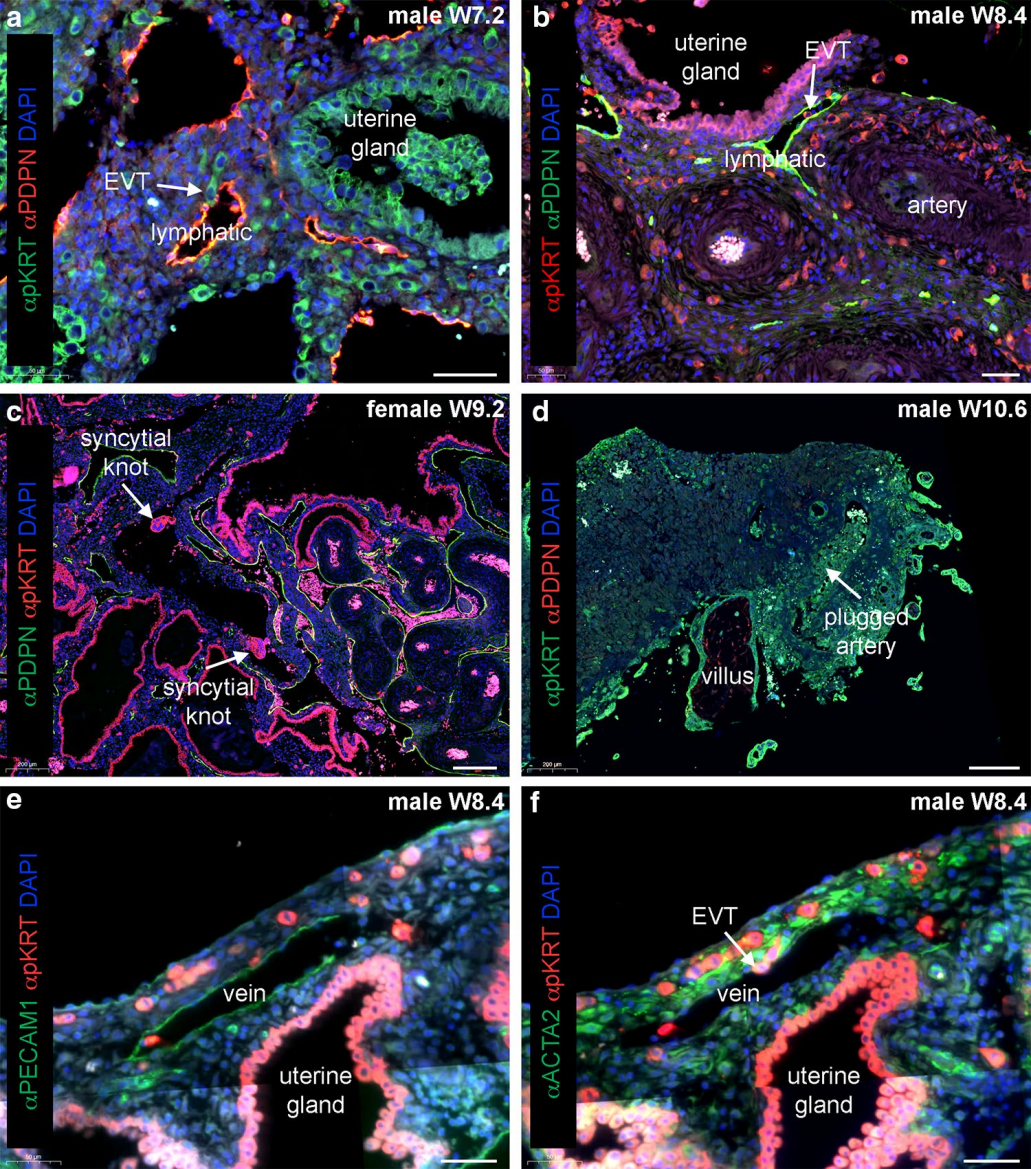
Endovenous and endolymphatic trophoblast invasion in normal pregnancies. Sections from first trimester decidua basalis (gestational age 7.2–10.6 weeks). **a** Immuno-double staining for pan cytokeratin (pKRT, green) and podoplanin (PDPN, red) with nuclear counterstain (DAPI, blue). **b** Immuno-double staining for pKRT (red) and PDPN (green) with DAPI (blue). White arrows in **a, b** depict endolymphatic trophoblasts invading lymphatic vessels. **c** Immuno-double staining for pKRT (red) and PDPN (green) with DAPI (blue). White arrows depict syncytial knots within a uterine vein. **d** Immuno-double staining for pKRT (green) and PDPN (red) with DAPI (blue). White arrow depicts a plugged artery. **e, f** Immuno-double staining for **e** pKRT (red) and PECAM1 (green) with DAPI (blue) and an adjacent section **f** with immuno-double staining for ACTA2 (green) and pKRT (red) with DAPI (blue). White arrow depicts endovenous trophoblasts invading a vein. EVT, extravillous trophoblast. Scale bars represent 50 µm in **a, b, e, f** and 200 µm in **c, d**

In the decidua during pregnancy, the arteries are responsible for blood transport to the decidua as well as the placenta, while veins and lymphatics are responsible for the transport of blood and lymph fluid from the decidua and the placenta (for blood). As described above, extravillous trophoblasts are known to invade uterine spiral arteries starting at around mid-first trimester (Brosens et al. 2002; Harris 2010; Weiss et al. 2016). However, prior to invasion into arteries, before week 6 of pregnancy, interstitial trophoblasts migrate deep into the uterus and reach the myometrium. At week 5, already 10% of all uterine veins in the placental bed are invaded by endovenous trophoblasts (He et al. 2017). Additionally, extravillous trophoblasts were observed to penetrate into relatively large caliber veins and lymphatics, but not arteries (Fig. 2a, b) (He et al. 2017; Moser et al. 2017; Windsperger et al. 2017). This takes place at a time in development when there is even no flow of maternal plasma through the intervillous space of the placenta.

The invading extravillous trophoblasts enter the maternal veins (endovenous trophoblast) and lymphatics (endolymphatic trophoblast) and may well be washed into the maternal blood stream. Thus, they could be responsible for the presence of fetal cells and DNA in maternal blood from week 6 onward (Chamley et al. 2011, 2014; Sarker et al. 2014). Functionally, this may be of importance to trigger maternal immune tolerance and the mother’s response to the developing embryo in the uterus. Starting with the second trimester of pregnancy, maternal blood flows through the intervillous space of the placenta. The normal turnover of the syncytiotrophoblast results in release of corpuscular particles from the syncytiotrophoblast (Goswami et al. 2006; Huppertz 2010; Huppertz et al. 1998b; Ikle 1964; Johansen et al. 1999; Redman and Sargent 2000). The multinucleated particles, called syncytial knots, enter the venous system and can be detected in uterine veins behind the placenta (Fig. 2c). The syncytial knots are mostly engulfed by macrophages in the lungs and thus are very rarely detected in peripheral blood. In very rare cases, circulating syncytial knots may cause local embolism in the lungs (Delmis et al. 2000).

Interestingly, the function of trophoblast invasion into uterine veins has been ignored for decades now. At the same time, the necessity and presence of connecting uterine veins to the intervillous space has never been questioned and already described by Ramsey (1959). Only very recently, invasion of endovenous trophoblast into uterine veins has been described (He et al. 2017; Moser et al. 2017; Windsperger et al. 2017). Different from arteries, for veins there is no need to transform the vessel wall as there is little vasoconstriction anyway. For veins, it seems to be sufficient to just invade such vessels and connect them to the intervillous space.

The function of the invasion of lymphatics is still not clear. Two possibilities are discussed:

1. Since endolymphatic trophoblast has been described very early in pregnancy, a function in transporting trophoblasts into the maternal circulation may be discussed. As described above, such embryonic cells may trigger the maternal immune tolerance to accept the semi-allograft within the uterus.
2. Trophoblast invasion may be much less restricted as hypothesized so far. Until now, trophoblast invasion was discussed to be restricted to just invasion of spiral arteries within the uterus, while all other structures did not seem to be target of the invasive capacity of extravillous trophoblast. This picture has changed now. It seems as if extravillous trophoblasts invade any luminal structure in the placental bed: glands, arteries, veins and lymphatics. There may not be a restriction to a specific type of structure; these cells may simply invade into all structures available along their invasive pathway.

### Endoglandular trophoblast invasion

**Fig. 3:**
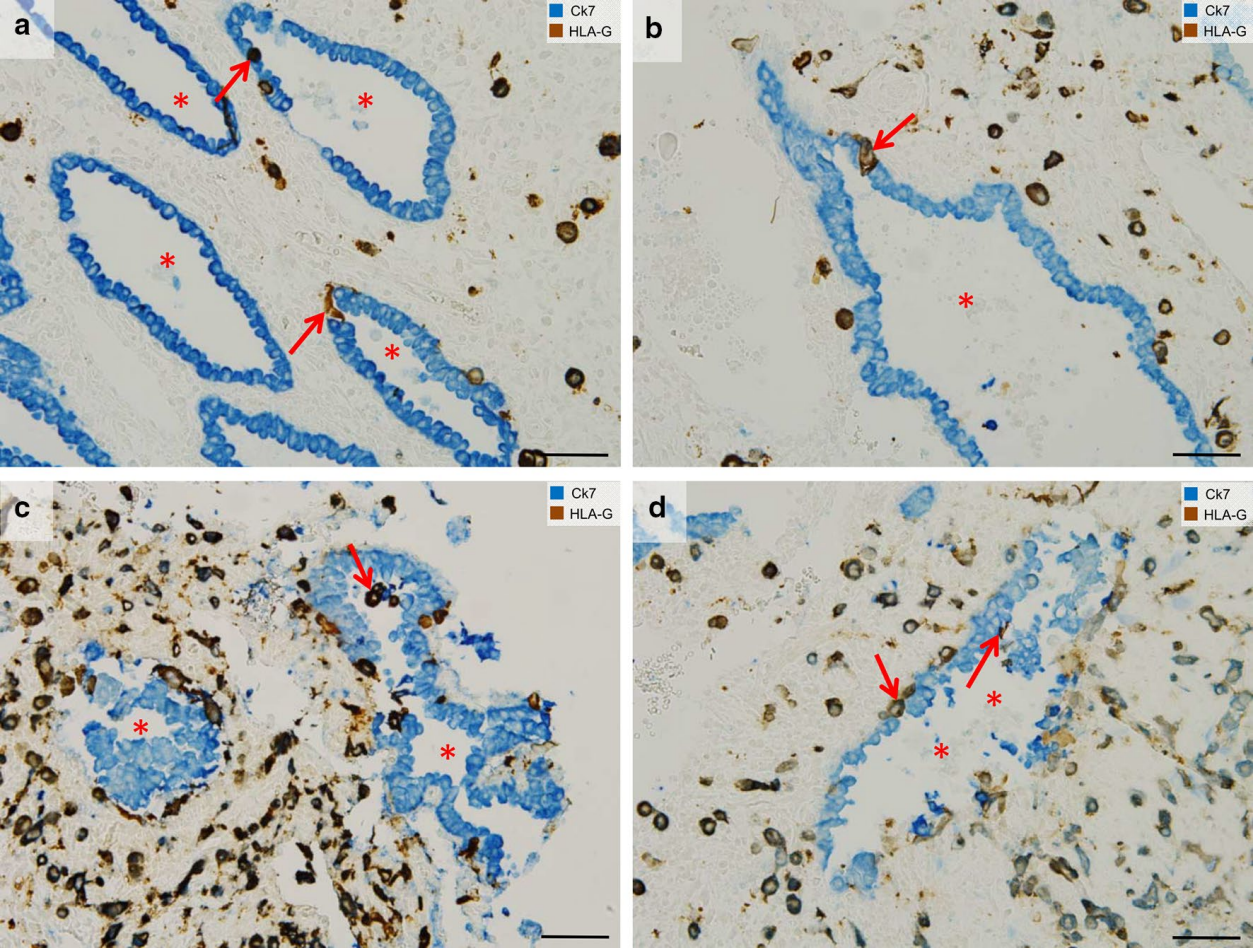
Endoglandular trophoblast invasion. Sections from the first trimester decidua basalis (gestational age 7–8 weeks). **a**–**d** Immuno-double staining for cytokeratin 7 (blue) and HLA-G (appears brown/dark violet) without nuclear counterstain. **a, b** Endoglandular trophoblasts (arrows) replace the epithelium of uterine glands (lumen of gland: asterisk). **c** Endoglandular trophoblasts are also situated in the glandular lumen. **d** Epithelium of invaded glands often appears disintegrated. Endoglandular trophoblasts, arrows; lumen of gland, asterisk. Scale bars represent 50 µm

In the last years, the close interaction between invading extravillous trophoblasts and uterine glands has been identified (Moser and Huppertz 2017; Moser et al. 2010, 2011, 2015). Very early in pregnancy, extravillous trophoblasts migrate toward the glandular epithelium on top of the glandular basal lamina, replace glandular epithelial cells by endoglandular trophoblasts (Fig. 3a, b, d) and invade into uterine glands (Fig. 3c, d). This seems to be the way how uterine glands are opened and connected toward the intervillous space of the placenta. Now, glandular secretion products can be released into the intervillous space. This was proved in situ by extensive assessment of sections of first trimester placental and placental bed specimens and in vitro with a specifically developed model system of early placenta/decidua confrontation co-culture (Moser et al. 2010, 2015).

Quantitative analysis of trophoblast invasion into vessels and glands of the placental bed revealed significantly more replacement of epithelial cells in glands compared to endothelial cells in blood vessels during the first trimester of pregnancy. Accumulation of detached glandular epithelial cells within the lumen of uterine glands occurs repeatedly in areas of strong trophoblast invasion. It is tempting to speculate that paracrine factors, secreted by interstitial and endoglandular trophoblasts, are responsible for the disintegration of the glandular epithelium (Moser et al. 2015).

A first connection between uterine glands and the implanting blastocyst is already visible at the time of implantation (Moser and Huppertz 2017; Moser et al. 2015). Hence, invasion into uterine glands is the very first step of trophoblast invasion in humans. This allows histiotrophic nutrition of the embryo to start as early as implantation takes place. Feeding of the early embryo by “uterine milk”, i.e., secretion products of uterine glands has already been shown in other mammals such as the horse (Allen and Stewart 2001). During the first trimester of pregnancy, endoglandular invasion and erosion of uterine glands occurs primarily at the margin of the developing placenta, supporting the lateral growth of the placenta (Moser and Huppertz 2017). Endoglandular trophoblasts express the pro-invasive proteins matrix metalloproteinase 1 (MMP1) (Weiss et al. 2016), matrix metalloproteinase 9 (MMP9) (Moser et al. 2015) and integrin β1 (ITGB1) (Moser et al. 2018).

### Extravillous trophoblasts migrate toward the cervix

Recently, HLA-G-positive extravillous trophoblasts have been retrieved from the endocervical canal and used for non-invasive prenatal testing (Bolnick et al. 2016a, b). These authors have shown that it is possible to collect extravillous trophoblasts from the cervix with a brush and separate trophoblasts from maternal cells for downstream analysis of fetal DNA, RNA, proteins and others (Drewlo and Armant 2017). Interestingly, retrieval of such cells can be performed from gestation week 5–20; afterward the cells can no longer be found and retrieved (Moser et al. 2018).

Two possible routes of how extravillous trophoblasts may reach the uterine cavity and subsequently the cervix have been discussed (Moser et al. 2018)

1. First, at the margin of the developing placenta interstitial trophoblasts may penetrate the uterine epithelium from the side of the connective tissue of the uterus, replace the uterine epithelium from the basal side and finally reach the uterine cavity (Moser et al. 2018).
2. Second, endoglandular trophoblasts may invade uterine glands at the margin of the developing placenta. These glands may still open toward the uterine cavity and thus the endoglandular trophoblasts are flushed together with the glandular secretion products into the uterine cavity (Moser et al. 2018).

The migratory potential of extravillous trophoblasts has already been proven by Billington (1966) and it is likely that—as soon as they reach the uterine cavity—they simply migrate from the uterine cavity toward the cervix (Billington 1966). This new retrieval method of extravillous trophoblasts from the cervix may open new strategies of noninvasive prenatal testing or even diagnosis.

## First insight into alterations of the migratory routes of extravillous trophoblast in pathological pregnancies

### Altered trophoblast invasion into uterine vessels in recurrent spontaneous abortions is accompanied by vascular changes in the decidua

Recurrent spontaneous abortion (RSA) is defined by the occurrence of three or more consecutive miscarriages before 20 weeks of gestation. In cases of idiopathic RSA, the role of arterial trophoblast invasion has historical roots but still remains controversial (Ball et al. 2006; Michel et al. 1990; Sebire et al. 2002). In the light of the recent findings on trophoblast invasion, showing the versatile functional routes of trophoblast invasion also into glands, veins and lymphatic vessels in the first trimester decidua, a critical re-evaluation is highly warranted. Indeed, a recently published analysis of extravillous trophoblast interaction with decidual arteries, veins and lymphatics revealed abnormalities in vascular invasion merely in non-arterial vessels (Windsperger et al. 2017). In detail, decidua basalis RSA tissues showed a lower ratio of invaded veins and lymphatics versus the total number of vessels, when compared to healthy controls with age-matched gestational ages. Interestingly, this phenomenon was particularly pronounced in lymphatics (Windsperger et al. 2017).

So far, it is not clear whether this pathological alteration points toward an RSA-associated placental phenotype characterized by compromised function of extravillous trophoblasts or is provoked by a dysregulated uterine environment. It was recently shown that tissues of decidua basalis of RSA cases generally contain more vessels (arteries, veins and lymphatics) than healthy controls (Windsperger et al. 2017). On one hand, this finding contrasts previous scientific reports showing no differences in vessel density between cases and controls or showing even fewer vessels with larger circumferences in decidua basalis of abortions (Plaisier et al. 2009; Vailhé et al. 1999). On the other hand, Quenby et al. (2009) identified an increased blood vessel density in the secretory endometrium of non-pregnant woman, diagnosed with RSA (Quenby et al. 2009). Along those lines, Plaisier et al. (2009) highlighted elevated levels of angiogenic factors in both secretory endometrium and decidua basalis samples of miscarriage patients. Hence, it seems reasonable that an augmented angiogenesis during the menstrual cycle causes an increased vessel density in the endometrium as well as decidua in RSA patients and may thus exhaust the capacity of extravillous trophoblasts to invade into luminal structures of the uterus. Therefore, the differentiated description of vascularization patterns in endometrial and decidual tissue of RSA patients may help to decipher the yet unclear picture of idiopathic recurrent pregnancy loss.

### Extravillous trophoblasts in tubal pregnancies

**Fig. 4:**
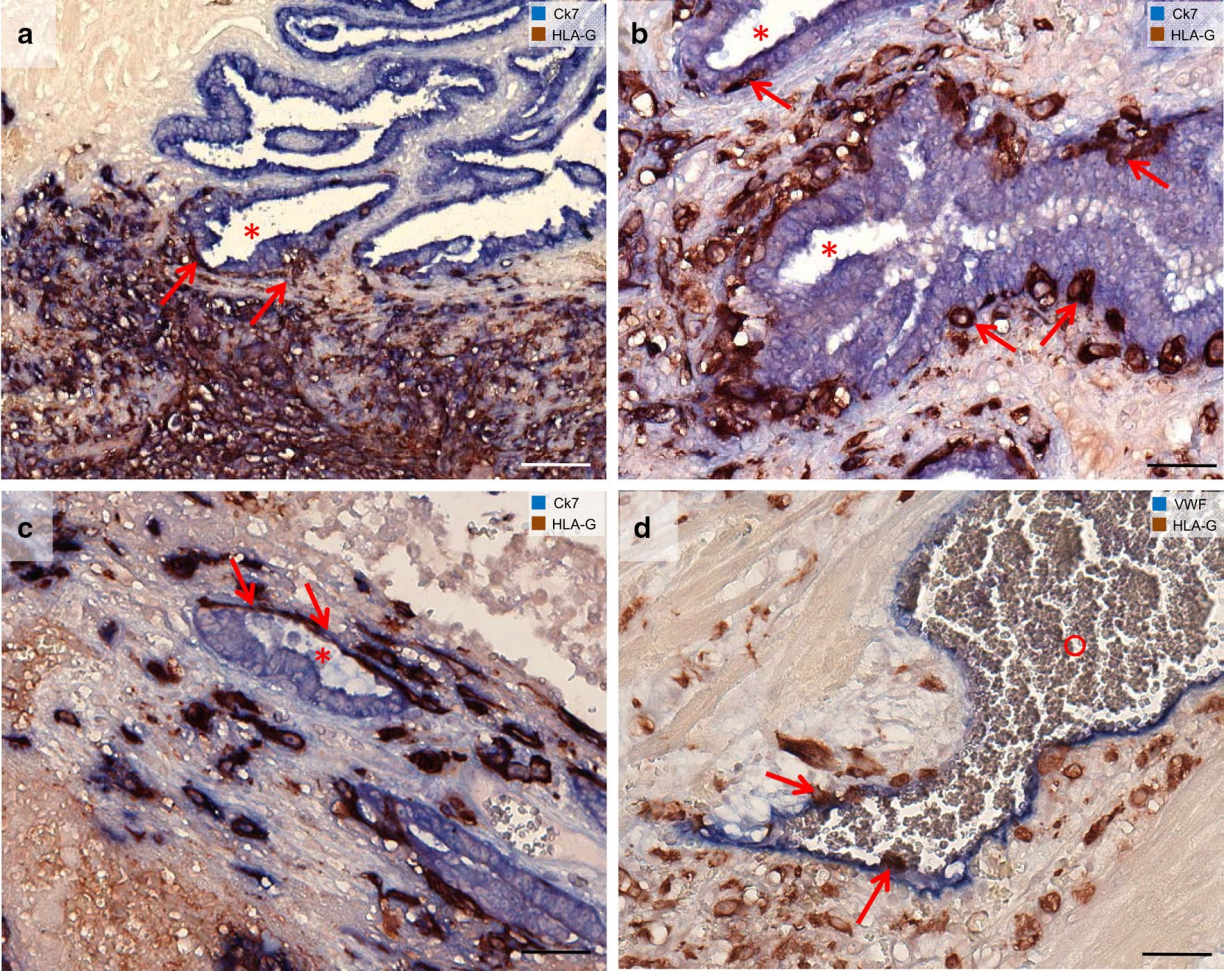
Invasion of extravillous trophoblasts in epithelial structures and vessels in tubal pregnancies. Sections from tubal pregnancies (**a**–**c**) are immuno-double stained for cytokeratin 7 (blue) and HLA-G (appears brown/dark violet) without nuclear counterstain. **a** Overview. Extravillous trophoblasts (arrows) invade the lamina propria of the mucosal folds (asterisk). Trophoblasts can be found beneath the epithelium. **b, c** The epithelium of the mucosal folds is penetrated by extravillous trophoblasts (arrows) from the basal side. **d** Extravillous trophoblasts are visualized with immuno-double staining for vWF (blue) and HLA-G (brown). Single endovascular trophoblasts (**d**, arrows) are situated in the lumen of the vessel (circle). Scale bars represent 100 µm in **a** and 50 µm in **b, c, d**

Implantation sites within the uterus and the fallopian tube are similar in composition and cell density of the tissues comprising the surface epithelium and the lamina propria of the tubal or uterine wall containing connective tissue, glandular and surface epithelium, trophoblast cells and lymphocytes (Kemp et al. 2011). However, the cellular morphology of invading extravillous trophoblasts within the placental bed differs between normal uterine and viable tubal pregnancies (Hammer 2011; Kemp et al. 2002). Emmer et al. (2002) observed that blood-filled lacunae and endovascular trophoblasts are present in tubal pregnancies. This might indicate (functional) remodeling of tubal blood vessels by trophoblasts (Emmer et al. 2002). In addition, in tubal pregnancies extravillous trophoblasts seem to invade toward the epithelium of mucosal folds coming from the lamina propria (Fig. 4). They can be found beneath the epithelium and seem to replace the epithelium from the basal side (Fig. 4a–c). Extravillous trophoblasts in tubal pregnancies have already been described to invade vessels (Li et al. 2015; Randall et al. 1987), while single cells can also be found in the lumen of the vessels (Fig. 4d).

## Conclusions

Extravillous trophoblasts invade into much more luminal structures in the maternal decidua than was known until today. It seems as if extravillous trophoblasts invade into each luminal structure that is present along their invasive pathway. Hence, it seems as if there is no restriction of trophoblast invasion in terms of specificity of invaded structures. The elucidation of alterations of trophoblast invasion in pregnancy pathologies has only just begun. The following years will tell how many of the alterations have been missed so far and may explain pathologies such as recurrent spontaneous abortions and fetal growth restriction.
